# Cardiovascular Risk Prediction by the American Diabetes Association Risk-Assessment Tool and Novel and Traditional Cardiovascular Risk Factors in Young Adults With Type 1 Diabetes

**DOI:** 10.7759/cureus.22574

**Published:** 2022-02-24

**Authors:** Isabel Inácio, Teresa Azevedo, José Luís Martins, Ana Margarida M Balsa, Rosa Dantas, Márcia Alves, Isabel Albuquerque, Joana Guimarães

**Affiliations:** 1 Endocrinology Department, Centro Hospitalar do Baixo Vouga, Aveiro, PRT; 2 Cardiology Department, Centro Hospitalar do Baixo Vouga, Aveiro, PRT; 3 Endocrinology Department, Centro Hospitalar de Trás-os-Montes e Alto Douro, Vila Real, PRT; 4 Nutrition Department, Centro Hospitalar do Baixo Vouga, Aveiro, PRT

**Keywords:** cardiovascular risk (cvr), myocardial infarction (mi), cardiovascular disease, american diabetes association risk-assessment tool: 10-year risk for diabetes complications, cardiovascular risk factor, traditional cardiovascular risk factors, novel cardiovascular risk factors, young adult, cardiovascular risk assessment, type 1 diabetes mellitus (t1d)

## Abstract

Introduction: Cardiovascular disease is an important cause of morbidity and mortality in individuals with type 1 diabetes (T1D). The American Diabetes Association (ADA) has the ADA risk-assessment tool for cardiovascular risk (CVR) prediction in individuals with T1D. This study aims to evaluate the prevalence of novel and traditional cardiovascular risk factors (CVRF) and the CVR by the ADA risk-assessment tool: 10-year risk for diabetes complications in young adults with T1D.

Methods: Cross-sectional observational study of T1D individuals aged 18-40 years and T1D duration ≥1 year. The ADA risk-assessment tool was applied to predict CVR.

Results: 75 individuals, 61.3% male, with a median age of 30 (26.0-36.0) and 13.0 (6.0-20.0) years of T1D duration. Hypertension was found in 16% of individuals and dyslipidemia in 75.0%. 21.3% were active smokers, 30.7% sedentary, and 42.7% were at least overweight. Most individuals had a 10-year risk <1% for all complications except myocardial infarction (MI). In individuals who were outside the honeymoon period (T1D duration ≥ 5 years), most had a 10-year risk <1% for all complications except MI and amputation. Non-traditional CVRF homocysteine, apolipoprotein B, apolipoprotein B/apolipoprotein A1 ratio, magnesium, and vitamin D correlated with the ADA risk-assessment tool. 10-year risk for MI ≥1% was significantly more frequent in men.

Conclusion: To our knowledge, this is the first study to apply the ADA risk-assessment tool: 10-year risk for diabetes complications in T1D. Young adults with T1D have a worrying prevalence of CVRF and show suboptimal control. Most individuals with T1D duration ≥1 year have an estimated 10-year risk <1% for all complications, except for MI.

## Introduction

Cardiovascular disease (CVD) is an important cause of morbidity and mortality in individuals with type 1 diabetes (T1D) [1,2]. However, cardiovascular risk (CVR) reduction strategies have been extrapolated in large part from the knowledge about type 2 diabetes (T2D), despite the longer disease duration in T1D and the differences in pathophysiology [3]. The typical phenotype of patients with T1D (young people with normal body mass index (BMI)) and low prevalence of other cardiovascular risk factors (CVRF) is changing, due to the growing epidemic of obesity and risk of weight gain associated with glycemic control [3-5].

There are no recommended CVR prediction calculators for widespread use in T1D [3]. SCORE (Systematic COronary Risk Evaluation) is widely used in Europe [6,7]. This calculator uses age, sex, systolic blood pressure (SBP), total cholesterol (TC), high-density lipoprotein (HDL) cholesterol, and smoking habits. It advocates that there are qualifiers/modifiers that should be investigated to improve the CVR estimate and that individuals with diabetes have high or very high CVR, but some young people may have a lower CVR [6]. However, SCORE only calculates the risk of fatal CVD.

The 2022 American Diabetes Association (ADA) guidelines suggest the 10-year risk calculator of the first CVD event called The American College of Cardiology/American Heart Association (AHA) atherosclerotic cardiovascular disease (ASCVD) risk calculator (Risk Estimator Plus) [8,9]. It uses age, sex, race, SBP, diastolic blood pressure (DBP), TC, HDL cholesterol, low-density lipoprotein (LDL) cholesterol, history of diabetes (yes/no), smoking, and use of antihypertensive medication, statin, or aspirin. However, this calculator estimates the risk for ages ≥40 years and does not include the type or duration of diabetes or complications, such as albuminuria [8,10]. Although some variability in calibration exists in various subgroups, including by sex, race, and diabetes, the overall risk prediction does not differ in those with or without diabetes, validating the use of risk calculators in people with diabetes [8].

Another option for estimating CVR in individuals with T1D, mentioned by the ADA and AHA, is the ADA risk-assessment tool [3,10]. This calculator uses age, gender, height, weight, BMI, smoking habits (in the last month), weekly exercise (</≥ 150 minutes per week on average), daily use of aspirin, SBP and DBP, use of antihypertensive medication, TC, HDL, triglycerides (TG); medical history of MI, heart failure, atrial fibrillation, heart disease, kidney failure or dialysis, stroke, peripheral arterial disease or peripheral vascular disease, blindness or amputation because of diabetes; diabetes history (T1D or T2D, diabetes with unknown type, prediabetes, without diabetes); age at diagnosis and HbA1c [10]. Although this calculator does not include the presence of albuminuria like the ASCVD Risk Estimator Plus, it contemplates parameters not included in the other calculators mentioned above, such as the presence of renal failure or dialysis and other complications, duration, and type of diabetes; height, weight, BMI, weekly exercise, TG, medical history of MI, heart failure, atrial fibrillation, age at diagnosis and HbA1c.

The present study aims to assess the prevalence of CVRF, calculate the CVR using the ADA risk-assessment tool: 10-year risk for diabetes complications and to evaluate the association with other novel/non-traditional and traditional CVRF and CVR qualifiers not included in the ADA risk-assessment tool in a population of young adults with T1D. This study was previously presented as a meeting abstract at the Portuguese Society of Diabetology annual meeting 2018 (14º Congresso Português de Diabetes) on March 9-11, 2018.

## Materials and methods

This observational cross-sectional study included adults aged between 18 and 40 years with T1D duration ≥1 year, followed at the Endocrinology Department of Centro Hospitalar do Baixo Vouga (CHBV) between July 2017 and August 2018. Clinical data were collected, and laboratory analyses and electrocardiogram (ECG) were requested. Transthoracic echocardiography (TTE) was requested from individuals who had hypertension (HT). HT was considered if previous diagnosis or under antihypertensive medication. According to the recommendations of the European Society of Cardiology (ESC)/European Association for the Study of Diabetes (EASD) [6] and ESC/European Atherosclerosis Society (EAS) [7] and in the absence of reference values in these recommendations according to the reference values of the laboratory of our center, dyslipidemia was defined if at least one parameter related to the lipid profile was altered (TC >190 mg/dl, LDL cholesterol ≥100 mg/dl, non-high-density lipoprotein (non-HDL) cholesterol ≥130 mg/dL, TG ≥150 mg/dL, apolipoprotein B/apolipoprotein A1 (ApoB/A1) ratio >0.7, lipoprotein (a) (Lp(a)) >120 nmol/L), or under antidyslipidemic medication.

The CVR estimation was performed using the ADA risk-assessment tool: 10-year risk for diabetes complications [10]. With this calculator, the 10-year risk for the following complications is obtained: myocardial infarction (MI), stroke, chronic kidney disease (CKD), blindness due to diabetic retinopathy (DR), and amputation. Informed consent was obtained from all individuals. The study was authorized by the Ethics Committee of CHBV and was carried out in conducted in accordance with The Declaration of Helsinki.

Statistical analysis was performed using SPSS program Released 2015 (IBM SPSS Statistics for Windows, Version 23.0. Armonk, NY: IBM Corp). The data are reported as the median and percentiles 25 and 75 (P25-75) for continuous variables or as percentages and absolute numbers for categorical variables. Fisher’s exact and Mann-Whitney tests and correlation between variables were used. A value of p <0.05 was considered statistically significant.

## Results

We included 75 individuals (61.3% male), with a median age of 30 (26.0-36.0) years and 13.0 (6.0-20.0) years of T1D duration. The largest age group was that of later young adults (31-40 years, 49.3%). The general characteristics and prevalence of CVRF are shown in Table 1. Overall, dyslipidemia was found in 75% of individuals (Table 1), with hypercholesterolemia occurring in 39.2% and hypertriglyceridemia in 6.7%. Considering the CVR categories and the current LDL targets [8,9], one (11.1%) of the nine individuals at moderate risk had LDL above the target (≥100 mg/dL). Of the 44 individuals at high risk, 86.4% (38) had LDL ≥70 mg/dL. Of the 22 subjects at very high risk, all had LDL ≥55 mg/dL.

**Table 1:** Clinical and biochemical characteristics of young adults with type 1 diabetes. Data are presented as median (percentiles 25-75) or as % (n). BMI: body mass index; CHO: carbohydrate; DBP: diastolic blood pressure; HbA1c: hemoglobin A1c; HDL: high-density lipoprotein cholesterol; HR: heart rate; hs-CRP: high-sensitivity C-reactive protein; HT: hypertension; LDL: low-density lipoprotein cholesterol; non-HDL: non-high-density lipoprotein cholesterol; SBP: systolic blood pressure; TC: total cholesterol; TDID: total daily insulin dose; TG: triglycerides; WC: waist circumference; WHR: waist-hip ratio

Clinical and biochemical characteristics	
Male, % (n)	61.3% (46)
Age groups, % (n)	
18-25 years	24.0% (18)
26-30 years	26.7% (20)
31-40 years	49.3% (37)
Educational level, years in school	12.0 (12.0-15.0)
Educational level, % (n)	
No information	13.3% (10)
≤9 years	14.7% (11)
10-12 years	42.7% (32)
≥13 years	29.3% (22)
Work situation, % (n)	
No information	12.1% (9)
Employee/student	78.6% (59)
Unemployed	9.3% (7)
CHO counting, % (n)	40.0% (30)
TDID, UI/day	56.0 (44.0-70.0)
FreeStyle Libre flash glucose monitoring system, % (n)	6.7% (5)
HT, % (n)	16.0% (12)
Dyslipidemia, % (n)	75.0% (51)
Currently medicated	13.2% (9)
Not currently medicated	61.8% (42)
Smoking, % (n)	
Never smoked	76.0% (57)
Current smokers	21.3% (16)
Former smokers	2.7% (2)
Alcohol consumption, % (n)	
Never	42.7% (32)
Occasional consumption	42.7% (32)
Daily consumption	8.0% (6)
Physical activity, % (n)	
Never	30.7% (23)
Physically active	34.7% (26)
“Unstructured” physical activity	34.7% (26)
Anthropometric data	
BMI, kg/m^2 ^	24.4 (22.5-27.4)
Overweight/obesity (BMI ≥25.0 kg/m^2^), % (n)	42.7% (32)
Class I-III obesity (BMI ≥30.0 kg/m^2^), % (n)	10.6% (8)
WC, cm	83.5 (75.8-92.9)
Female, WC >80.0/>88.0 cm, % (n)	48.3% (14)/27.6% (8)
Male, WC >94.0/>102.0 cm, % (n)	26.1% (12)/8.7% (4)
WHR	0.82 (0.12-0.92)
Female, WHR ≥0.85	27.6% (8)
Male, WHR ≥0.90	45.7% (21)
Periodontal disease, % (n)	11.4% (5)
Mental illness, % (n)	10.7% (8)
Depression	6.7% (5)
Diabetes complications, % (n)	
Retinopathy	16.0% (12)
Nephropathy	9.3% (7)
Neuropathy	0% (0)
Cerebrovascular disease	0% (0)
Ischemic heart disease	0% (0)
Peripheral arterial disease	0% (0)
SBP, mmHg	127.0 (118.0-136.0)
DBP, mmHg	80.0 (73.0-89.0)
HR, bpm	78.0 (67.0-89.0)
Hemoglobin, g/dL	14.6 (13.6-16.1)
HbA1c, %	7.7% (7.1-8.8)
Fasting blood glucose, mg/dl	165.0 (104.0-216.0)
Uric acid, mg/dL	4.0 (3.3-5.1)
TC, mg/Dl	181.5 (161.5-201.0)
HDL, mg/dL	59.3 (47.0-72.9)
LDL, mg/dL	105.0 (85.0-128.0)
non-HDL, mg/dL	119.6 (100.4-139.3)
TG, mg/dL	79.0 (60.0-113.0)

Most individuals (81.3%) had HbA1c ≥7%. In addition to T1D, median number of other CVRF was 3.0 (2.0-5.0): 96% of individuals had at least one other CVRF, 78.7% had at least two other CVRF, and 58.7% had at least other three CVRF. Men had significantly higher levels of uric acid, TC/HDL ratio and homocysteine, and significantly lower levels of high-sensitivity C-reactive protein (hs-CRP), HDL, and Apo-A1 compared to women (Table 2).

**Table 2:** Distribution of biochemical characteristics of young adults with type 1 diabetes by sex. Data are presented as median (percentiles 25-75) and n. Mann-Whitney test was used. BNP: B-type natriuretic peptide; HDL: high-density lipoprotein cholesterol; hs-CRP: high-sensitivity C-reactive protein; TC: total cholesterol

	Male, median (P25-P75) (n)	Female, median (P25-P75) (n)	p value
BNP, pg/mL	5.5 (2.3-12.8) (44)	13.0 (5.0-18.0) (23)	0.030
Hemoglobin, g/dL	15.7 (14.5-16.3) (46)	13.5 (12.7-14.2) (25)	<0.001
hs-CRP, mg/dL	0.08 (0.02-0.46) (42)	0.35 (0.08-0.91) (24)	0.020
Uric acid, mg/dL	4.6 (3.9-5.6) (45)	4.3 (2.8-3.9) (25)	<0.001
HDL, mg/dL	54.1 (44.3-69.8) (46)	66.3 (50.5-79.1) (29)	0.008
TC/HDL ratio	3.4 (2.7-4.1) (46)	2.8 (2.5-3.0) (28)	0.009
Apolipoprotein A1, mg/dL	149.0 (127.0-174.0) (33)	177.0 (146.5-200.5) (21)	0.009
Creatinine, mg/dL	1.0 (0.9-1.1) (46)	0.8 (0.7-0.9) (27)	<0.001
Urea, mg/dL	37.0 (30.9-44.8) (46)	29.7 (28.2-34.4) (24)	0.020
Homocysteine, µmol/L	12.2 (8.8-19.7) (33)	9.4 (7.2-10.4) (21)	0.001

No patient had a previous diagnosis of arrhythmia or CVD (Table 1). However, of the 44 patients who underwent ECG, 18 (40.9%) had an abnormal ECG. Of these, seven (38.9%) had bradycardia and two (11.1%) tachycardia at rest. Left ventricular hypertrophy (LVH) electrocardiographic criteria were detected in three (16.7%) individuals, left atrial dilation in two (18.2%), cardiac rhythm changes in two, and isolated supraventricular extrasystole in one. Of the 12 patients with HT, eight underwent TTE. In these, changes in left ventricular function and/or structure were found in five (41.7%) patients. The individuals presented elevations in inflammatory plasma markers associated with CVD and alterations in other markers considered as a novel or non-traditional RF (Table 3). None of these markers referred to in Table 3 is incorporated in the ADA risk-assessment tool.

**Table 3:** Non-traditional cardiovascular risk factors in young adults with type 1 diabetes*. Data are presented as % (n). Fisher’s exact test was used. *cardiovascular risk factors not incorporated in the ADA risk-assessment tool: 10-year risk for diabetes complications. ApoA1: apolipoprotein A1; ApoB: apolipoprotein B; BNP: B-type natriuretic peptide; hs-CRP: high-sensitivity C-reactive protein; TC: total cholesterol; TG: triglycerides

	Total, % (n)	Male, % (n)	Female, % (n)	p value
hs-CRP >0,50 mg/dL (n=63)	33.3% (21)	25.6% (10)	45.8% (11)	0.110
Fibrinogen >350 mg/dL (n=66)	19.7% (13)	19.0% (8)	20.8% (5)	0.551
BNP ≥25/≥35 pg/mL (n=67)	9.0% (6)/1% (1.5)	6.8% (3)/0%(0)	13.0% (3)/4.3% (1)	0.406/0.343
Magnesium ≤1,80 mg/dL (n=70)	24.3% (17)	17.8% (8)	36.0% (9)	0.144
Vitamin D <20 ng/mL (n=75)	69.3% (52)	76.1% (35)	58.6% (17)	0.129
Homocysteine ≥15 µmol/L (n=54)	22.2% (12)	36.4% (12)	0% (0)	0.002
TC ≥190 mg/dL (n=74)	39.2% (29)	37.0% (17)	42.9% (12)	0.396
TG ≥150 mg/dL (n=75)	6.7% (5)	6.5% (3)	6.9% (2)	0.646
ApoB/A1 ratio >0,7 (n=54)	17.1% (7)	29.2% (7)	0.0% (0)	0.029
Lipoprotein(a) >120 nmol/L (n=54)	11.1% (6)	8.8% (3)	15.0% (3)	0.391

Cardiovascular risk (CVR)-ADA risk-assessment tool: 10-year risk for diabetes complications

**Figure 1:**
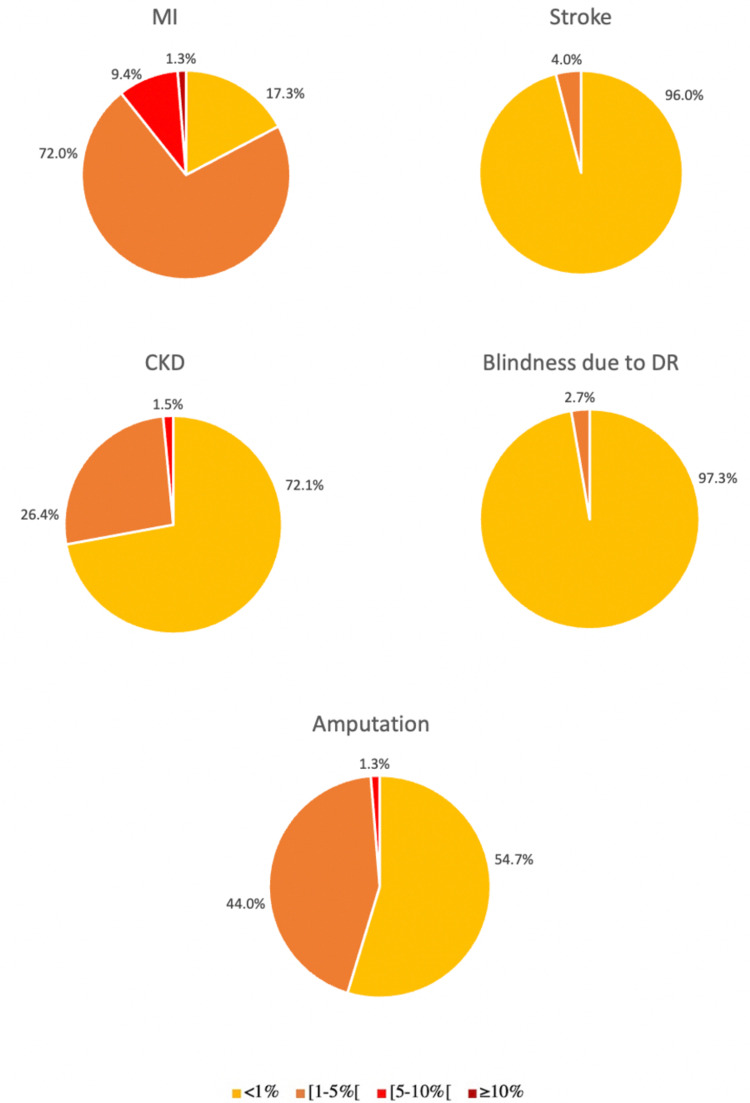
Cardiovascular risk prediction by the ADA risk-assessment tool: 10-year risk for diabetes complications in young adults with type 1 diabetes. “[” was used to indicate that the ranges “[1-5%[” and “[5-10%[” do not include the value of “5%” and “10%”, respectively. ADA: American Diabetes Association; MI: myocardial infarction; CKD: chronic kidney disease; DR: diabetic retinopathy

CVR calculated using the ADA risk-assessment tool was ≥1% for MI in 82.7% (62) of the individuals, for stroke in 4.0% (3), for CKD (excluding individuals with nephropathy) in 27.9% (19), for blindness due to DR in 2.7% (2), and for amputation in 45.3% (34) (Figure 1). For any complication, the 10-year risk ≥1% was found in 90.7% (68).

10-year risk ≥1% alone for MI was significantly more frequent in males (p=0.001), with no significant differences between genders for the remaining complications. Moderate-to-high-risk young adults with T1D for MI tended to have higher levels of uric acid, compared to low-risk young adults with T1D for MI (Table 4).

**Table 4:** Comparison between moderate-to-high risk versus low-risk young adults with type 1 diabetes for myocardial infarction according to the ADA risk-assessment tool. Data are presented as median (percentiles 25-75) and n. Mann-Whitney test was used. ApoA1: apolipoprotein A1; ApoB: apolipoprotein B; BNP: B-type natriuretic peptide; HDL: high-density lipoprotein cholesterol; hs-CRP: high-sensitivity C-reactive protein; MI: myocardial infarction; TC: total cholesterol; T1D: type 1 diabetes

	Moderate-to-high-risk young adults with T1D for MI, median (P25-P75) (n)	Low-risk young adults with T1D for MI, median (P25-P75) (n)	pvalue
BNP, pg/mL	6.5 (2.8-15.3) (58)	10.0 (6.0-15.5) (9)	0.390
Hemoglobin, g/dL	14.9 (13.9-16.2) (60)	13.6 (13.1-14.6) (11)	0.014
hs-CRP, mg/dL	0.12 (0.03-0.73) (56)	0.22 (0.01-0.66) (10)	0.886
Fibrinogen, mg/dL	292.0 (255.8-335.5) (56)	313.0 (265.5-333.8) (10)	0.648
Magnesium, mg/dL	1.97 (1.83-2.08) (59)	2.01 (1.87-2.09) (11)	0.622
Uric acid, mg/dL	4.1 (3.4-5.3) (59)	3.6 (3.0-3.9) (11)	0.056
TC/HDL ratio	3.1 (2.5-4.0) (62)	2.7 (2.5-2.8) (12)	0.086
Vitamin D, ng/mL	14.5 (10.5-19.8) (59)	16.3 (14.6-25.2) (11)	0.223
Creatinine, mg/dL	0.9 (0.8-1.0) (61)	0.8 (0.7-1.0) (12)	0.067
Urea, mg/dL	35.6 (29.9-41.9) (59)	29.3 (26.1-33.6) (11)	0.034
Homocysteine, µmol/L	10.5 (8.4-14.8) (46)	9.9 (8.1-10.3) (8)	0.408
ApoA1, mg/dL	159.0 (136.8-184.3) (46)	171.5 (124.5-204.0) (8)	0.503
ApoB/A1 ratio	0.43 (0.00-0.58) (46)	0.47 (0.45-0.56) (8)	0.462
Lipoprotein(a), nmol/L	17.0 (6.0-50.0) (47)	17.0 (6.0-25.0) (7)	0.573

**Figure 2:**
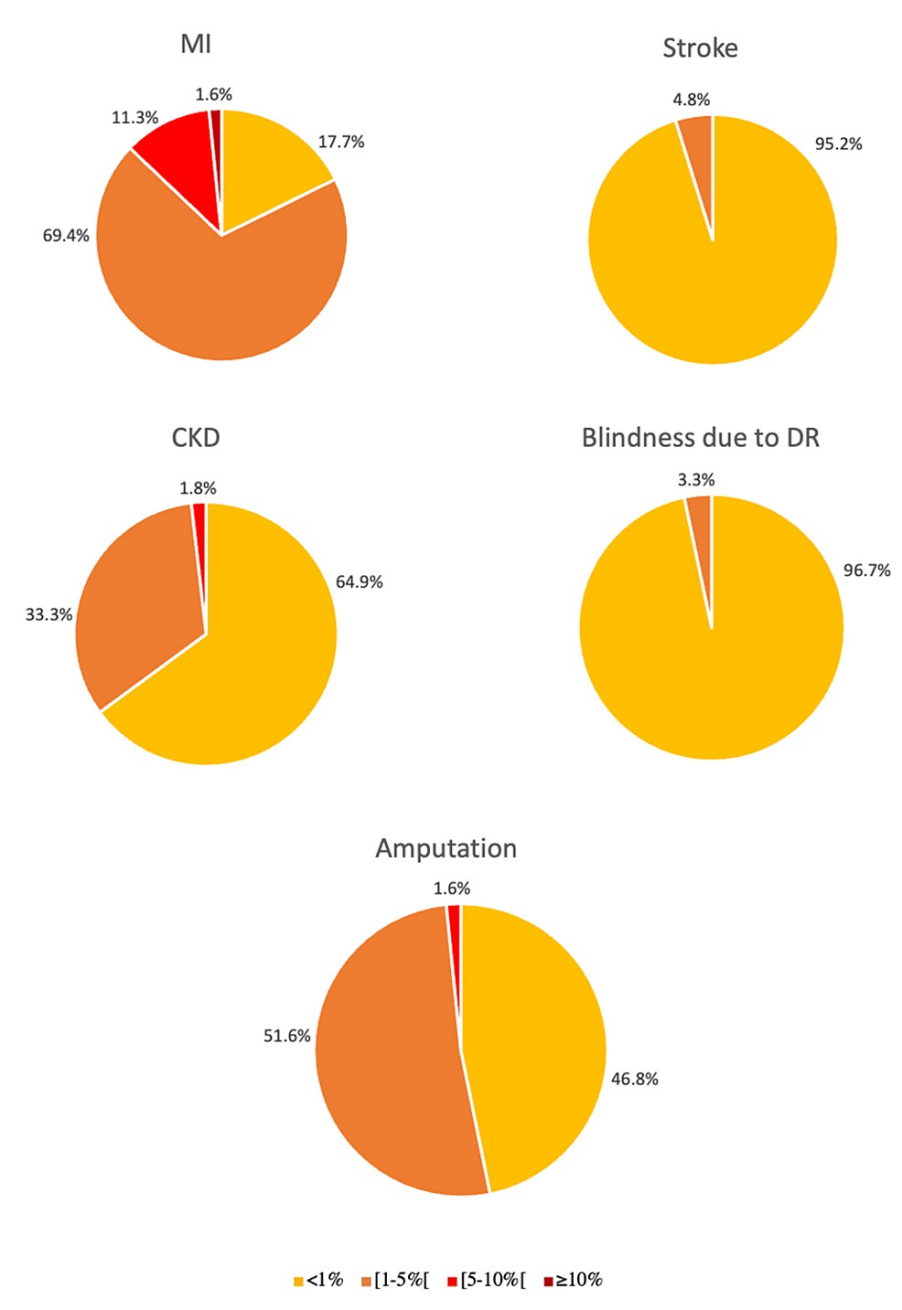
Cardiovascular risk prediction by the ADA risk-assessment tool: 10-year risk for diabetes complications in young adults with type 1 diabetes duration ≥5 years. “[” was used to indicate that the ranges “[1-5%[” and “[5-10%[” do not include the value of “5%” and “10%”, respectively. ADA: American Diabetes Association; MI: myocardial infarction; CKD: chronic kidney disease; DR: diabetic retinopathy

As individuals with T1D duration ≥1 year were included, some individuals could still be in the honeymoon period. Thus, we performed an analysis in which only patients who are unequivocally after the honeymoon period were included (T1D duration ≥5 years, n=62) (Figure 2). Although most patients had a higher 10-year risk for amputation, the remaining findings were similar.

Overall, the 10-year risk for MI showed an acceptable and positive correlation with uric acid (ρ (spearman’s rho)=0.458, p=0.001), hemoglobin (ρ=0.403, p=0.001), urea (ρ=0.388, p=0.001), creatinine (ρ=0.369, p=0.001), and homocysteine (ρ=0.334, p=0.001). It also presented a positive correlation, although weak with total daily insulin dose (ρ=0.239, p=0.045). The 10-year risk for CKD was acceptably correlated with waist circumference (WC; ρ=0.493, p=<0.001), waist-hip ratio (WHR; ρ=0.405, p = 0.001), ApoB (ρ=0.387, p=0.015) and ApoB/A1 ratio (ρ=0.356, p=0.010). With the levels of magnesium and vitamin D, a negative but weak correlation was found (ρ=−0.259, p=0.036; and ρ=−0.249, p=0.044, respectively). Regarding the 10-year risk for amputation, a positive and acceptable correlation was found with apolipoprotein B (ρ=0.420, p=0.006), and positive, however, weak with urea (ρ=0.249, p=0.044). There was a negative and acceptable correlation with albumin (ρ=−0.394, p=0.001). Also, with magnesium levels, the correlation found was negative and weak (ρ =−0.291, p=0.015). No statistically significant correlations were found with the 10-year risk for stroke and for blindness due to DR.

## Discussion

In the present study, we found prevalences between 16% and 75% for major modifiable CVRF (HT, dyslipidemia, smoking, overweight/obesity, and physical inactivity) in young adults with T1D. The prevalence of HT in our population (16%) is consistent with the previously described, although it is heterogeneous (11-59%) [3,4]. Dyslipidemia was found in 75% of patients. This prevalence agreed with that found in recent studies (67.5-72.5%), but these also included children and adolescents [11,12]. Most individuals have an LDL above the current recommended target [6,7], proving non-optimized control in young adults. Hypercholesterolemia was higher (39.2%) than previously described (7.4-15.6%) [11,13]. However, these studies included children up to 31 years old adults, and in our study, the age group from 31 to 40 years had a considerable representation (49.3%). Hypertriglyceridemia was found in 6.7%, similarly to previous studies (5-30%) [4,14].

There is little information on the prevalence or effects of smoking in T1D [3]. The prevalence described in T1D aged between 20 and 22 years was 34.0% in one study [15]. Active smoking was found in 21.3% of our population, which is relevant new data. Traditionally, people with T1D have normal weight, however, this has changed. The prevalence of obesity increased from 1% at the Diabetes Control and Complications Trial baseline to 31% at the Epidemiology of Diabetes Interventions and Complications year 12 [16]. In our study, a lower prevalence of obesity (10.6%) was found, with 32.1% being overweight and 30.7% sedentary. In our study, the majority (81.3%) had HbA1c higher than the target of 7%, which agrees with other studies [17-18]. This is a point to improve in the management of these individuals since the protective effects of controlling early blood glucose levels tend to persist for several decades with CVR reduction [3].

Data found in men seem to point to a more adverse CVR profile in this gender. However, CVD has been reported to equally affect women and men with T1D <40 years in the United Kingdom, and after 40 years, men are more affected than women [3,19]. Other studies reported higher CVD in adult women with T1D compared to men [11,20,21]. In our study, the apparently more unfavorable CVR profile in men can be justified by the high prevalence of dyslipidemia and smoking in men. Electrocardiographic and echocardiography changes were not previously known in 40.9% and 41.7%, respectively, some of which may be associated with an increased risk of CVD [6]. Furthermore, some subjects showed changes in novel and non-traditional CVRF, namely elevation of Lp(a), hs-CRP, homocysteine, fibrinogen and B-type natriuretic peptide, hypomagnesemia, and hypovitaminosis D, which can help to reclassify individuals or individually identify greater risk.

The ADA risk-assessment tool reclassified individuals as mostly low risk (<1%) for all complications except MI. Moderate-to-high-risk young adults with T1D for MI only tended to have higher levels of uric acid compared to low-risk young adults, with no statistically significant differences for the other non-traditional and traditional CVRF studied. We speculate that, as we included individuals with T1D duration ≥1 year, some individuals could still be in the honeymoon period. This could have an impact on HbA1c levels, because lower HbA1c levels may have resulted in a lower calculated CVR. Therefore, in a sub-analysis of 62 patients who are after the honeymoon period (T1D duration ≥ 5 years), most patients continued to be at low risk for all complications, except for MI and amputation in that the majority had moderate risk (Figure 2).

This calculator appears to be only differentiating for the risk categories for MI and eventually for amputation. However, these data can be justified by the fact that age is a major CVRF in the short- and medium term, which means that risk calculators may not be reliable in predicting CVR in the long term in young. One way around this disadvantage is to compare the CVR of an individual with T1D with the minimum CVR of individuals of the same age and sex without the disease [22]. Treatment, namely with aspirin or statin as the primary prevention in this age group, could be considered when the CVR was more than three times the minimum risk for age and sex [22].

The markers uric acid, homocysteine, hemoglobin, urea, and creatinine were associated with a higher 10-year risk for MI, which agrees with the literature [23-26]. Also, the increase in WC, WHR, ApoB, and ApoB/A1 ratio was associated with a higher 10-year risk of CKD. These data are expected since central obesity defined by WC increases the risk of CKD regardless of BMI [27]. Similarly, WHR is a significant predictor of proteinuria and low eGFR [28]. This was a single-center and observational cross-sectional study, which limits the extrapolation of data to other populations. Although the CVR calculator used is recommended by ADA, its accuracy in T1D is not clear [3], which may have contributed to the low reclassification of CVR for all complications, except for MI and amputation.

Nevertheless, our study presents as major strengths the fact that it evaluates the restricted age range of young adults with T1D, as many studies cover children and adolescents as well as adults; and the extensive evaluation of traditional and novel/non-traditional CVRF/CVR qualifiers. It contributes to a greater knowledge of the current prevalence of active smoking in young adults with T1D. A calculator recommended by the ADA was applied that allows the CVR assessment in young people with T1D (<40 years). Because it is a technology applied to diabetes that is accessible online and free, it can be a co-adjuvant tool in the therapeutic education in T1D.

## Conclusions

The prevalence of CVRF in our population of young adults with T1D presents worrying values and reveals suboptimal control. Since it is a population still without established macrovascular complications, our study shows that it is necessary to increase early preventive strategies. Novel or non-traditional CVR markers were significantly correlated with the ADA risk-assessment tool. To our knowledge, this is the first study to apply the ADA risk-assessment tool: 10-year risk for diabetes complications in T1D. Most subjects with T1D duration ≥1 year have a 10-year risk <1% for all diabetes complications except for MI. Most patients after the honeymoon period (T1D duration ≥5 years) have a 10-year risk <1% for all diabetes complications except for MI and amputation. Unfortunately, it is still required a CVR calculator with greater precision in T1D to clearly guide preventive and therapeutic measures in young adults with T1D. Future studies to increase knowledge of T1D-related CVD are warranted.
